# Identifying Clinical Predictors of Raised Intracranial Pressure in Pediatric Traumatic Brain Injury—A Multinational Initiative

**DOI:** 10.1177/2689288X251370703

**Published:** 2025-09-09

**Authors:** Noah Xin Ying Yeo, Hui Jun Zhou, Jan Hau Lee, Paula Caporal, Juan D. Roa G, Sebastián González-Dambrauskas, Jane Pei Wen Ng, Yoko Wong, Adriana Yock-Corrales, Yasser Kazzaz, Qalab Abbas, Shu-Ling Chong

**Affiliations:** ^1^ Duke-NUS Medical School, Singapore, Singapore.; ^2^ Singapore Clinical Research Institute, Singapore, Singapore.; ^3^ Children’s Intensive Care Unit. KK Women’s and Children’s Hospital, Singapore, Singapore.; ^4^ SingHealth Pediatrics Academic Clinical Programme, Duke-NUS Medical School, Singapore, Singapore.; ^5^ Johns Hopkins International Injury Research Unit, Health Systems Program, Department of International Health, Johns Hopkins Bloomberg School of Public Health, Baltimore, Maryland, USA.; ^6^ Red Colaborativa Pediátrica de Latinoamérica (LARed Network), Montevideo, Uruguay.; ^7^ Pediatric Neurocritical Care Unit Fundación Homi (hospital de la Misericordia), Universidad del Bosque, Bogotá, Colombia.; ^8^ Departamento de Pediatría Y Unidad de Cuidados Intensivos de Niños del Centro Hospitalario Pereira Rossell, Facultad de Medicina, Universidad de La República, Montevideo, Uruguay.; ^9^ KK Research Centre, KK Women’s and Children’s Hospital, Singapore, Singapore.; ^10^ Emergency Department, National Children’s Hospital “Dr. Carlos Saenz Herrera” CCSS, San José, Costa Rica.; ^11^ Department of Paediatrics, Ministry of National Guard Health Affairs, Riyadh, Saudi Arabia.; ^12^ College of Medicine, King Saud bin Abdulaziz University for Health Sciences, King Abdul Aziz Medical City, Jeddah, Saudi Arabia.; ^13^ King Abdullah International Medical Research Centre, King Abdul Aziz Medical City, Riyadh, Saudi Arabia.; ^14^ Departments of Pediatrics and Child Health, National Stadium Road, Aga Khan University Hospital, Karachi, Pakistan.; ^15^ Department of Emergency Medicine, KK Women’s and Children’s Hospital, Singapore, Singapore.; ^16^ SingHealth Paediatrics Academic Clinical Programme, Emergency Medicine Academic Clinical Programme, Duke-NUS Medical School, Singapore, Singapore.

**Keywords:** child, clinical predictors, monitoring, raised intracranial pressure, traumatic brain injury

## Abstract

Use of the intracranial pressure (ICP) monitor in pediatric traumatic brain injury (TBI) remains variable. Clinical prediction models of raised ICP have been reported in adult TBI but have not been validated in pediatric TBI. We aimed to investigate clinical predictors and derive a prediction model for raised ICP in pediatric patients with TBI. A real-world observational study was conducted among pediatric intensive care units from the Pediatric Acute & Critical Care Medicine Asian Network and Red Colaborativa Pediátrica de Latinoamerica. Children <18 years with moderate-to-severe TBI and who were hospitalized between 2014 and 2022 were included. We defined raised ICP as >20 mmHg. Multivariable logistic regression models were built to identify significant predictors for raised ICP, and performance was assessed using the area under the receiver operating characteristic curve (AUC). Among 706 pediatric patients, only 151 (21.4%) had ICP monitoring, and 75 (49.7%) were confirmed to have raised ICP. Mortality was 13.2%, 8.0%, and 4.0% for patients who did not receive ICP monitoring, those with raised ICP, and those with normal ICP, respectively (*p* = 0.037). A model predicting for raised ICP comprising sex, Glasgow Coma Scale motor score, leukocytosis, thrombocytopenia, and skull fracture on computed tomography performed with a sensitivity, specificity, and AUC of 56.0% (95% confidence interval [CI]: 44.8%−67.2%), 75.0% (95% CI: 65.3%−84.7%), and 73.7% (95% CI: 65.7%−81.6%), respectively. We report clinical predictors associated with raised ICP in pediatric TBI. The clinical prediction model was not sensitive, and future large-scale prospective studies should stratify predictors by specific intracranial pathologies.

## Introduction

Traumatic brain injury (TBI) can result in cerebral bleeding, edema, and raised intracranial pressure (ICP).^1,2^ The Brain Trauma Foundation (BTF) recommends ICP monitoring in severe pediatric TBI.^3^ The insertion of ICP monitoring involves technical challenges in the pediatric population because of their narrower ventricular systems.^4–6^ At the same time, these invasive procedures can cause infection and hemorrhagic complications.^7–9^ A previous study by the Pediatric Acute & Critical Care Medicine Asian Network (PACCMAN) found that children in low- to middle-income countries (LMIC) who suffered from moderate-to-severe TBI often did not receive ICP monitoring, and these children were more likely to have poor neurological functional outcomes.^10^ Furthermore, not all centers in high-income countries use ICP monitoring routinely, due to physician-dependent variability.^10,11^

Clinical predictors of raised ICP have been identified in adult TBI. The Benchmark Evidence from South American Trials: Treatment of Intracranial Pressure (BEST:TRIP), a multicenter randomized controlled trial, reported similar clinical outcomes in patients with TBI managed with ICP monitoring compared with those managed with a clinical protocol based on computed tomography (CT) imaging findings, Glasgow Coma Scale (GCS) motor score, and pupillary asymmetry.^12^ Another retrospective cohort study reported that older age, pupillary fixation, signs of significant head trauma, and the need for intubation were significant predictors of raised ICP.^13^ However, evidence on clinical predictors of raised ICP has been sparse for the pediatric population. A study on the feasibility of early identification of raised ICP in pediatric TBI found that diffuse axonal injury was the only independent variable that was significantly associated with the development of raised ICP.^14^ Hence, we aimed to identify independent predictors of raised ICP in children with moderate-to-severe TBI and to build a clinical prediction model for raised ICP.

## Materials and Methods

### Study design

We performed a secondary analysis of a multinational observational study between two pediatric intensive care unit networks, namely the PACCMAN and the Red Colaborativa Pediátrica de Latinoamerica (LARed Network) across Asia, Europe, and Latin America. In the main study, clinical outcomes were described among a prospective cohort of children with moderate-to-severe TBI who received different hyperosmolar agents.^15^ In this secondary analysis, we used retrospective data from 2014 to 2017, and prospective data from 2018 to 2022 from PACCMAN sites. LARed sites collected prospective data from 2021 to 2022. We included patients under 18 years old hospitalized for moderate-to-severe TBI (GCS = 3–13). We excluded patients who arrived at participating sites ≥24 h post-injury or who had missing data on mortality and Pediatric Cerebral Performance Category (PCPC) at discharge. Ethics approval was first obtained from the coordinating hospital in Singapore (SingHealth Centralized Institutional Review Board CIRB 2018/2457) and then from each of the participating sites.

### Study variables

Variables were established by the core study team in Singapore and participating principal investigators to guide data collection. Data were collected in a standardized electronic form (REDCap, Vanderbilt University) with fields defined *a priori*. We collected information on patient demographics, mechanism of injury, time to arrival, laboratory findings, and CT findings. Collaborating sites were categorized based on their 2016 Sociodemographic Index (SDI), which is region-specific, accounts for educational attainment, income per person, and total fertility rate.^16^ We grouped low-income countries, LMIC, and middle-income countries as low- to middle-income SDI countries. Multiple trauma was defined as head injury plus at least one of thoracic injury, abdominal injury, or long bone injury. Thrombocytopenia was defined as a platelet count <150 × 10^9^/L.^17^ Leukocytosis was defined as a leukocyte count >11.0 × 10^9^/L.^18^ Dysnatremia was defined as sodium values <135 or >145 mmol/L.^19^ Coagulopathy was defined as prothrombin time >15 s, partial thromboplastin time >40 s, or an international normalized ratio >1.2.^20^ We also collected data on the use of hyperosmolar therapy and the need for mechanical ventilation within 24 h of hospital admission. Hyperosmolar therapy was defined as the use of 3% hypertonic saline or 20% mannitol within the first 24 h of hospital admission. Raised ICP was defined as >20 mmHg within the first 24 h of hospital admission. We did a sensitivity analysis for ICP >25 mmHg in order to make comparisons at different thresholds.^21^ Decisions on ICP management, including the use of hyperosmolar agents, were based on clinician practices and hospital guidelines.

We also obtained inpatient mortality and PCPC on discharge.^22,23^ PCPC was graded at baseline and upon discharge and categorized into (1) good; (2) mild disability; (3) moderate disability; (4) severe disability; (5) vegetative state/coma; and (6) death.^22,23^ Among patients who survived, a PCPC score of 3–5 was considered as poor functional outcome.^10^ Patients with in-hospital mortality were separately described.

### Statistical analysis

The sample was stratified by ICP status (raised ICP, no raised ICP, and without ICP monitoring). Categorical variables were analyzed with Pearson’s chi-squared test. Post hoc analysis with Bonferroni correction was used for pairwise comparison between the groups.^24^ Statistical significance was taken at *p* < 0.05 unless a stricter *p* < 0.017 was used for ad hoc pairwise comparisons.

Multivariable logistic regression (MLR) applying the least absolute shrinkage and selection operator^25^ approach identified clinical variables that were significantly associated with raised ICP in 151 patients with ICP monitoring. Potential independent variables were first examined using univariate analysis and then subject to MLR according to statistical significance in univariate analysis (*p* < 0.2), known pilot data, published literature, or clinical consensus reached previously.^19,26^ Both unadjusted and adjusted odds ratio (aOR) were presented with the corresponding 95% confidence intervals (CIs). The performance of the prediction models for raised ICP was illustrated in receiver operating characteristic (ROC) curves and quantified with area under the curve (AUC), sensitivity, and specificity. The statistical significance was set at *p* < 0.05 unless otherwise stated for Bonferroni adjustment, where *p* < 0.017 was used.

The data were analyzed using SPSS v28 (IBM Corp. Released 2021. IBM SPSS Statistics for Macintosh, Version 28.0. Armonk, NY: IBM Corp., Chicago) and STATA v18 (StataCorp. 2023. Stata Statistical Software: Release 18. College Station, TX: StataCorp LLC).

## Results

### Presenting characteristics of study population

**FIG. 1.**
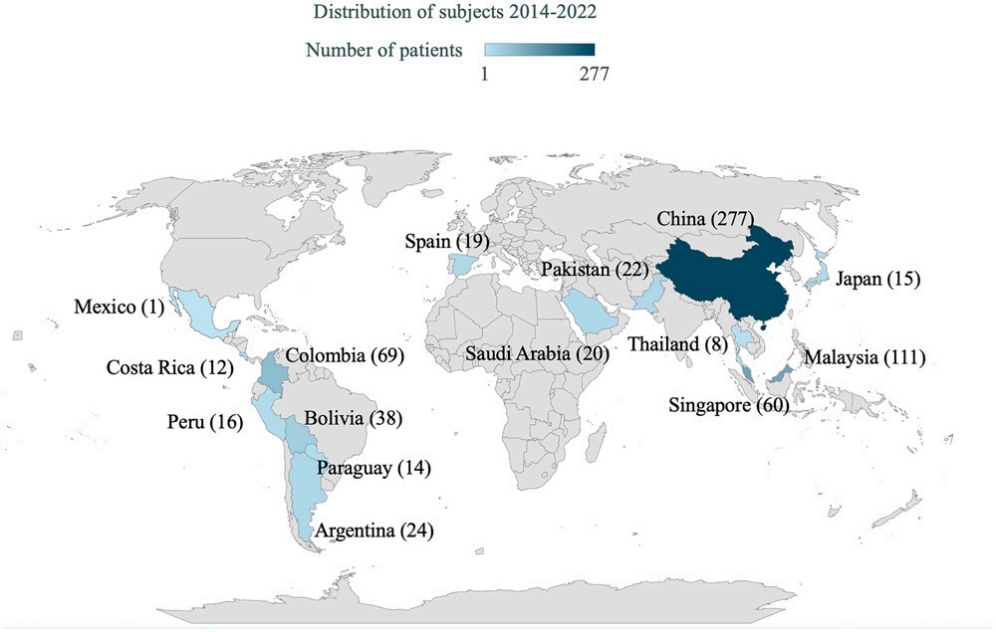
World map showing countries of participating sites. Hospitals from China, Japan, Malaysia, Pakistan, Saudi Arabia, Thailand, and Singapore were from the Pediatric Acute & Critical Care Medicine Asian Network (PACCMAN). Hospitals from Argentina, Bolivia, Colombia, Costa Rica, Mexico, Paraguay, Peru, and Spain were from Red Colaborativa Pediátrica de Latinoamerica (LARed Network). Created from Microsoft Excel for Mac version 16.77.1.

**FIG. 2.**
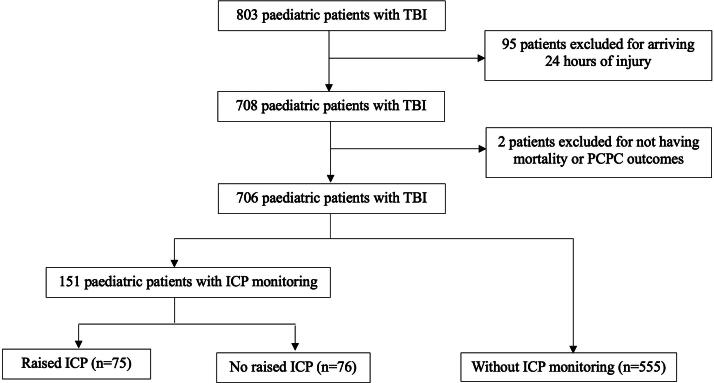
Participants’ flowchart for comparison of ICP status. ICP monitoring, intracranial pressure monitoring; PCPC, Pediatric Cerebral Performance Category; TBI, traumatic brain injury.

Among 706 pediatric patients analyzed, 513 (72.7%) were from PACCMAN (China, Japan, Malaysia, Pakistan, Saudi Arabia, Thailand, and Singapore), while 193 (27.3%) were from the LARed Network (Argentina, Bolivia, Colombia, Costa Rica, Mexico, Paraguay, Spain, and Peru) (Fig. 1). A total of 151 (21.4%) children received ICP monitoring; 75 (49.7%) had raised ICP (>20 mmHg) (Fig. 2). Among 706 patients, the majority (452, 64.0%) were males, with a median age of 4 years (interquartile range [IQR]: 2–9) (Table 1). Road traffic collisions accounted for 331 injuries (46.9%), followed by 294 falls (41.6%). There were 344 patients (48.7%) who presented with multiple trauma (Table 1). There were 441/706 (62.4%) with a total GCS 3–8 and 268/706 (38.0%) with a motor GCS <4.

**Table 1.** Patient Demographics and Clinical Features in the Study Population

Variable	Raised ICP, *n* = 75	No raised ICP, *n* = 76	No ICP monitoring, *n* = 555	*p* Value (three-way)	*p* Value raised ICP versus no raised ICP	*p* Value raised ICP versus no ICP monitoring	*p* Value no raised ICP versus no ICP monitoring
Median age (IQR)	6.0 (3.0–9.0)	5.0 (2.0–10.8)	4.0 (2.0–9.0)	0.305	0.513	0.140	0.539
Missing, *n* (%)	0	0	1 (0.18)	—	—	—	—
Male sex	42 (56.0)	55 (72.4)	355 (64.0)	0.111	0.042	0.160	0.203
Low-to-middle SDI	35 (46.7)	22 (28.9)	242 (43.6)	0.038^*^	0.026	0.616	0.016^*^
Transported by ambulance	65 (86.7)	64 (84.2)	435 (78.4)	0.169	0.818	0.127	0.293
Missing, *n* (%)	0 (0.0)	0 (0.0)	2 (0.4)	—	—	—	—
Median time to arrival, hours (IQR)	5.0 (1.0–8.0)	2.0 (0.3–5.0)	3.0 (1.0–8.0)	0.072	0.210	0.042	0.149
Mechanism of injury				0.153	0.995	0.135	0.178
Road traffic collision	41 (54.7)	41 (53.9)	249 (44.9)				
Fall	24 (32.0)	25 (32.9)	245 (44.1)				
Other	10 (13.3)	10 (13.2)	61 (11.0)				
Median presenting total GCS (IQR)	7.0 (5.0–10.0)	7.0 (5.0–9.0)	7.0 (5.0–11.0)	0.075	0.249	0.025	0.489
Missing, *n* (%)	1 (1.3)	0 (0.0)	0 (0.0)	—	—	—	—
Median presenting GCS motor (IQR)	4.0 (3.0–5.0)	4.0 (2.0–4.0)	4.0 (2.0–5.0)	0.051	0.115	0.742	0.015^*^
Missing, *n* (%)	1 (1.3)	0 (0.0)	0 (0.0)	—	—	—	—
Multiple trauma^a^	38 (50.7)	39 (51.3)	267 (48.1)	0.818	0.935	0.683	0.601
Long bone fracture	19 (25.3)	20 (26.3)	111 (20.0)	0.291	—	—	—
Intrathoracic injury	25 (33.3)	25 (32.9)	194 (35.0)	0.913	—	—	—
Intraabdominal injury	13 (17.3)	7 (9.2)	76 (13.7)	0.375	—	—	—

Analysis of variance test or Kruskal–Wallis test was used for three-way analysis depending on normality. Student *t* test or Mann–Whitney *U* test is used for two-way analysis depending on normality. Categorical variables were analyzed with Pearson’s chi-squared test.

^a^ Multiple trauma was defined as a head injury and at least one thoracic injury, abdominal injury, or long bone injury.

^*^ *p* < 0.05 was considered statistically significant (Bonferroni corrections were conducted for *post hoc* analysis, with a *p* value <0.017 being considered significant unless specified).

GCS, Glasgow Coma Scale; ICP, intracranial pressure; IQR, interquartile range; SDI, Social Demographic Index.

### CT findings, laboratory results, and interventions instituted

More patients with raised ICP developed midline shifts on CT compared with those without raised ICP and those who did not receive ICP monitoring (34.7% vs. 32.9% vs. 19.5%, *p* = 0.002) (Table 2). The three most common abnormalities were skull fractures (410/706, 58.1%), cerebral edema (207/706, 29.3%), and subdural hemorrhage (202/706, 28.6%) (Table 2). Patients with no raised ICP had significantly higher median leukocyte counts compared with patients with raised ICP and patients without ICP monitoring (19.3 × 10^9^/L [IQR: 16.2–23.8 × 10^9^/L] vs. 15.8 × 10^9^/L [IQR: 11.5–20.0 × 10^9^/L] vs. 15.9 × 10^9^/L [IQR: 11.8–20.7 × 10^9^/L], *p* ≤ 0.001) (Table 3). Patients with raised ICP had significantly lower median platelet counts than those with no raised ICP and no ICP monitoring (250.0 × 10^9^/L [IQR: 176–327.0 × 10^9^/L] vs. 318.5 × 10^9^/L [IQR: 250.0–378.0 × 10^9^/L] vs. 281.0 × 10^9^/L [IQR: 212.0–359.0 × 10^9^/L], *p* = 0.002) (Table 3). Children with raised ICP were more likely to receive hyperosmolar therapy compared with those who did not receive ICP monitoring, and those who did not have raised ICP (84.0% vs. 64.3% vs. 59.2%, *p* ≤ 0.001) (Table 4). Children with raised ICP and those who went for neurosurgical intervention were more likely to have ICP monitoring than those without raised ICP (88.0% vs. 76.3%, *p* = 0.034) (Table 4). Majority of patients with ICP monitoring were monitored with external ventricular drains in both the groups with raised ICP and those without raised ICP (54.7% vs. 46.1%, *p* = 0.298) (Table 4). Among patients with ICP monitoring, 25 (16.6%) received barbiturate therapy, and 58 (38.4%) had electroencephalogram monitoring (Table 4).

**Table 2.** Computed Tomography Findings in the Study Population

Parameters	Raised ICP, *n* = 75	No raised ICP, *n* = 76	No ICP monitoring, *n* = 555	*p* Value (three-way)	*p* Value raised ICP versus no raised ICP	*p* Value raised ICP versus no ICP monitoring	*p* Value no raised ICP versus no ICP monitoring
Midline shift on CT, *n* (%)	26 (34.7)	25 (32.9)	108 (19.5)	0.002^*^	0.863	0.005^*^	0.010^*^
Missing, *n* (%)	0 (0.0)	1 (1.3)	21 (3.8)	—	—	—	—
Skull fracture on CT, *n* (%)	50 (66.7)	36 (47.4)	324 (58.4)	0.052	0.017	0.170	0.068
Missing, *n* (%)	0 (0.0)	1 (1.3)	19 (3.4)	—	—	—	—
Subarachnoid hemorrhage, *n* (%)	37 (49.3)	30 (39.5)	120 (21.6)	<0.001^*^	0.252	<0.001^*^	0.001^*^
Missing, *n* (%)	0 (0.0)	1 (1.3)	19 (3.4)	—	—	—	
Subdural hemorrhage, *n* (%)	28 (37.3)	30 (39.5)	144 (25.9)	0.018^*^	0.7438	0.058	0.019
Missing, *n* (%)	0 (0.0)	1 (1.3)	19 (3.4)	—	—	—	
Epidural hematoma, *n* (%)	17 (22.7)	20 (26.3)	125 (22.5)	0.798	—	—	—
Missing, *n* (%)	0 (0.0)	1 (1.3)	19 (3.4)	—	—	—	
Intracerebral/intraparenchymal/intraventricular, *n* (%)	23 (30.7)	23 (30.3)	129 (23.2)	0.261	—	—	—
Missing, *n* (%)	0 (0.0)	1 (1.3)	19 (3.4)	—	—	—	
Hematoma (cerebrum/cerebellum), *n* (%)	19 (25.3)	14 (18.4)	115 (20.7)	0.605	—	—	—
Missing, *n* (%)	0 (0.0)	1 (1.3)	19 (3.4)	—	—	—	
Diffuse axonal injury, *n* (%)	5 (6.7)	11 (14.5)	66 (11.9)	0.275	—	—	—
Missing, *n* (%)	0 (0.0)	1 (1.3)	19 (3.4)	—	—	—	
Cerebral edema, *n* (%)	28 (37.3)	27 (35.5)	152 (27.4)	0.144	—	—	—
Missing, *n* (%)	0 (0.0)	1 (1.3)	19 (3.4)	—	—	—	
Pneumocephalus, *n* (%)	14 (18.7)	17 (22.4)	88 (15.9)	0.388	—	—	—
Missing, *n* (%)	0 (0.0)	1 (1.3)	19 (3.4)	—	—	—	
Brain stem injury/uncal herniation, *n* (%)	5 (6.7)	11 (14.5)	44 (7.9)	0.143	—	—	—
Missing, *n* (%)	0 (0.0)	1 (1.3)	19 (3.4)	—	—	—	
Undetermined, *n* (%)	0 (0.0)	0 (0.0)	6 (1.1)	0.429	—	—	—
Missing, *n* (%)	0	1 (1.3)	19 (3.4)	—	—	—	

Categorical variables were analyzed with Pearson’s chi-squared test.

^*^ *p* < 0.05 was considered statistically significant (Bonferroni corrections were conducted for *post hoc* analysis, with a *p* value <0.017 being considered significant unless specified).

CT, computed tomography; ICP, intracranial pressure.

**Table 3.** Laboratory Investigations in the Study Population

Variable	Raised ICP, *N* = 75	No raised ICP, *N* = 76	No ICP monitoring, *N* = 555	Total, *N* = 706	*p* Value (three-way)	*p* Value raised ICP versus no raised ICP	*p* Value raised ICP versus no ICP monitoring	*p* Value no raised ICP versus no ICP monitoring
Median leukocyte count (IQR) (×10^9^/L)	15.8 (11.5–20.0)	19.3 (16.2–23.8)	15.9 (11.8–20.7)	16.3 (12.0–21.0)	<0.001*	<0.001*	0.646	<0.001*
Missing, *n* (%)	0 (0.0)	0 (0.0)	6 (1.1)	6 (0.8)				
Median platelets (IQR) (×10^9^/L)	250.0 (176.0–327.0)	318.5 (250.0–378.0)	281.0 (212.0–359.0)	281.0 (212.3–359.0)	0.002*	<0.001*	0.014*	0.067
Missing, *n* (%)	0 (0.0)	0 (0.0)	6 (1.1)	6 (0.8)				
Median prothrombin time (IQR)	14.1 (13.1–18.2)	14.3 (13.0–17.0)	14.0 (12.7–16.0)	14.0 (12.7–16.2)	0.516	0.910	0.358	0.426
Missing, *n* (%)	5 (6.7)	1 (1.3)	21 (3.8)	27 (3.8)				
Median partial thromboplastin time (IQR)	32.8 (27.6–36.0)	31.8 (28.8–37.7)	31 (26.7–36.5)	31.2 (27.0–36.3)	0.260	0.756	0.325	0.154
Missing, *n* (%)	3 (4.0)	0 (0.0)	21 (3.8)	24 (3.4)				
Median international normalized ratio (IQR)	1.2 (1.1–1.5)	1.2 (1.1–1.3)	1.2 (1.1–1.3)	1.2 (1.1–1.3)	0.666	0.823	0.426	0.603
Missing, *n* (%)	6 (8.0)	2 (2.6)	54 (9.7)	62 (8.8)				
Median initial sodium (meq/L) (IQR)	138.0 (136.6–141.0)	138.8 (136.0–140.0)	139.0 (136.8–141.0)	139.0 (136.6–141.0)	0.511	0.441	0.894	0.247
Missing, *n* (%)	0 (0.0)	0 (0.0)	5 (0.9)	5 (0.7)				

Analysis of variance test or Kruskal–Wallis test was used for three-way analysis depending on normality. Student *t* test or Mann–Whitney *U* test is used for two-way analysis depending on normality.

ICP, intracranial pressure; IQR, interquartile range.

**Table 4.** Interventions, Mortality, and Functional Outcomes in the Study Population

Parameters	Raised ICP, *n* = 75	No raised ICP, *n* = 76	No ICP monitoring, *n* = 555	*p* Value (three-way)	*p* Value raised ICP versus no raised ICP	*p* Value raised ICP versus no ICP monitoring	*p* Value no raised ICP versus no ICP monitoring
ICP monitoring method, *n* (%)							
External ventricular drain	41 (54.7)	35 (46.1)	—	—	0.298	—	—
Subdural	7 (9.3)	2 (2.6)	—	—	—	—	—
Parenchymal bolt	15 (20.0)	20 (26.3)	—	—	—	—	—
Others	1 (1.3)	1 (1.3)	—	—	—	—	—
Missing, *n* (%)	11 (14.7)	18 (23.7)	—	—	—	—	—
Hyperosmolar therapy	63 (84.0)	45 (59.2)	357 (64.3)	<0.001^*^	0.001^*^	0.001^*^	0.385
Missing, *n* (%)	0 (0.0)	0 (0.0)	42 (7.6)	—	—	—	—
Intubation within 24 h	72 (96.0)	72 (94.7)	408 (73.5)	<0.001^*^	0.713	<0.001^*^	<0.001^*^
Missing, *n* (%)	0 (0.0)	0 (0.0)	1 (0.2)				
Barbiturate therapy	15 (20.0)	10 (13.2)	—	—	0.258	—	—
Missing, *n* (%)	0 (0.0)	0 (0.0)	—	—	—	—	—
Temperature control (°C)							
≥35	53 (70.7)	48 (63.2)	—	—	0.415	—	—
≥34	1 (1.3)	5 (6.6)	—	—	—	—	—
≥33	2 (2.7)	2 (2.6)	—	—	—	—	—
≥32	0 (0.0)	1 (1.3)	—	—	—	—	—
<32	19 (25.3)	20 (26.3)	—	—	—	—	—
Missing, *n* (%)	0 (0.0)	0 (0.0)	—	—	—	—	—
Head up 30°	69 (92.0)	72 (94.7)	—	—	0.499	—	—
Missing, *n* (%)	0 (0.0)	0 (0.0)	—	—	—	—	—
Electroencephalogram	26 (34.7)	32 (42.1)	—	—	0.347	—	—
Missing, *n* (%)	0 (0.0)	1 (1.3)	—	—	—	—	—
Neurosurgical intervention anytime during admission	61 (81.3)	56 (73.7)	—	—	0.170	—	—
Missing, *n* (%)	9 (12.0)	10 (13.2)	—	—	—	—	—
Decompressive craniectomy	24 (32.0)	17 (22.4)	—	—	0.194	—	—
Missing, *n* (%)	5 (6.7)	6 (7.9)	—	—	—	—	—
If neurosurgical intervention is performed, subsequent monitoring of ICP	66 (88.0)	58 (76.3)	—	—	0.034^*^	—	—
Missing, *n* (%)	5 (6.7)	6 (7.9)	—	—	—	—	—
Evacuation of intracranial bleed	20 (26.7)	22 (28.9)	—	—	0.712	—	—
Missing, *n* (%)	5 (6.7)	6 (7.9)	—	—	—	—	—
Elevation of depressed skull fracture	6 (8.0)	3 (3.9)	—	—	0.301	—	—
Missing, *n* (%)	5 (6.7)	6 (7.9)	—	—	—	—	—
If neurosurgical intervention, others	2 (2.7)	4 (5.3)	—	—	0.404	—	—
Missing, *n* (%)	5 (6.7)	6 (7.9)	—	—	—	—	—
In-hospital mortality, *n* (%)	6 (8.0)	3 (4.0)	73 (13.2)	0.037^*^	0.302	0.211	0.030
Missing, *n* (%)	0 (0.0)	0 (0.0)	0 (0.0)	—	—	—	—
Poor PCPC^a^	30/68 (44.1)	23/69 (33.3)	96/478 (20.1)	<0.001^*^	0.125	0.002^*^	0.315

Analysis of variance test or Kruskal–Wallis test was used for three-way analysis depending on normality. Student *t* test or Mann–Whitney *U* test is used for two-way analysis depending on normality. Categorical variables were analyzed with Pearson’s chi-squared test.

^a^ Poor PCPC was defined as a score of moderate disabilities (3), severe disabilities (4), and coma (5). Patients with in-hospital mortality were excluded from the analysis of PCPC outcomes.

^*^ *p* < 0.05 was considered statistically significant (Bonferroni corrections were conducted for *post hoc* analysis, with a *p* value <0.017 being considered significant unless specified).

ICP, intracranial pressure; PCPC, Pediatric Cerebral Performance Category.

### Mortality and functional outcomes

The overall in-hospital mortality rate in our cohort was 11.6% (82/706) (Table 4). Children who did not receive ICP monitoring had the highest mortality rate (13.2%), followed by those with raised ICP (8.0%) and those without raised ICP (4.0%) (*p* = 0.037) (Table 4). After excluding children who died (*n* = 82), those with poor PCPC at baseline (*n* = 4), and those without any recorded PCPC on discharge (*n* = 5), 149/615 (24.2%) patients suffered poor PCPC outcomes. Children with raised ICP had the highest proportion with poor PCPC (44.1%), followed by those without raised ICP (33.3%) and those who did not receive ICP monitoring (20.1%) (*p* < 0.001) (Table 4).

### Derivation and performance of the clinical prediction model for raised ICP

**FIG. 3.**
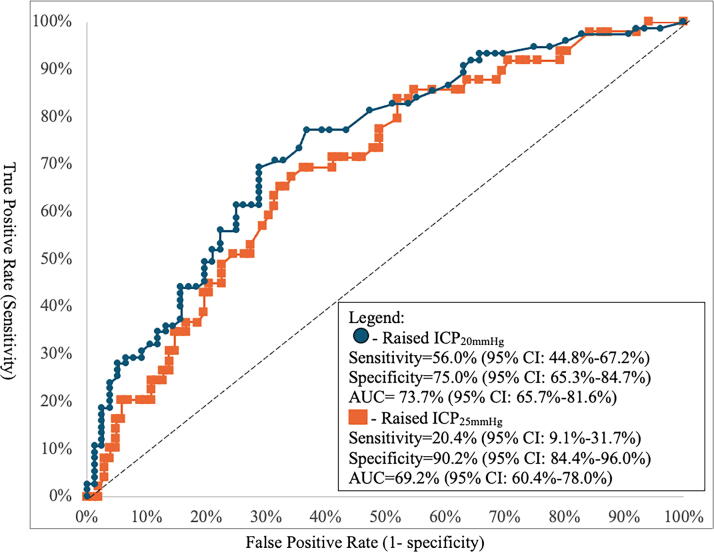
Predictive performance of receiver operating characteristic (ROC) curves derived from multivariable models at two thresholds for raised ICP at >20 mmHg (Raised ICP_20mmHg_) versus >25 mmHg (Raised ICP_25mmHg_). AUC, area under the curve; CI, confidence interval; ICP, intracranial pressure.

Sex, time to arrival, GCS motor score, leukocytosis, thrombocytopenia, and skull fracture were entered into the MLR. With raised ICP defined as >20 mmHg, leukocytosis was found to be associated with lower odds for raised ICP (aOR = 0.14, 95% CI: 0.03–0.67, *p* = 0.013) (Table 5). The model predicting ICP_20mmHg_ achieved a sensitivity of 56.0% (44.8 − 67.2%), a specificity of 75.0% (65.3–84.7%), and an overall performance of AUC being 73.7% (65.7–81.6%) (Fig. 3).

**Table 5.** Multivariable Logistic Regression Predicting for Raised Intracranial Pressure at Thresholds >20 mmHg and >25 mmHg

	Multivariable logistic regression model for raised ICP defined as ICP >20 mmHg (*n* = 151)				Multivariable logistic regression model for raised ICP defined as ICP >25 mmHg (*n* = 151)			
	Unadjusted OR with 95% CI	*p* Value	Adjusted OR with 95% CI	*p* Value	Unadjusted OR with 95% CI	*p* Value	Adjusted OR with 95% CI	*p* Value
Male sex	0.49 (0.25–0.96)	0.037*	0.51 (0.24–1.05)	0.068	0.49 (0.25–0.99)	0.059	0.49 (0.23–1.01)	0.052
Time to arrival (hours)	1.07 (1.00–1.15)	0.041*	1.06 (0.98–1.13)	0.140	1.08 (1.01–1.15)	0.033*	1.08 (1.01–1.16)	0.032*
GCS motor (<4)	0.77 (0.40–1.46)	0.416	0.85 (0.42–1.71)	0.643	1.59 (0.80–3.16)	0.187	1.65 (0.80–3.38)	0.173
Leukocytosis^a^	0.11 (0.02–0.49)	0.004*	0.14 (0.03–0.67)	0.013*	0.50 (0.18–1.38)	0.178	0.62 (0.21–1.83)	0.391
Skull fracture on CT	2.22 (1.15–4.29)	0.017*	1.89 (0.92–3.86)	0.082	1.67 (0.83–3.39)	0.152	1.54 (0.74–3.22)	0.249
Thrombocytopenia^b^	2.92 (1.07–7.99)	0.037*	1.64 (0.55–4.89)	0.375	—	—	—	—

^a^ Leukocytosis was defined as a leukocyte count >11.0 × 10^9^/L.

^b^ Thrombocytopenia was defined as a platelet count <150 × 10^9^/L. Thrombocytopenia was not selected in the multivariable logistic regression for ICP_25mmHg_.

CT, computed tomography; CI, confidence interval; GCS, Glasgow Coma Scale; ICP, intracranial pressure; OR, odds ratio.

With raised ICP defined as ≥25 mmHg, time to arrival was associated with higher odds (aOR = 1.08, 95% CI: 1.01–1.16, *p* = 0.032) (Table 5). Using the threshold of ICP_25mmHg_, the specificity of the MLR model increased to 90.2% (84.4 − 96.0%), while the sensitivity decreased to 20.4% (9.1 − 31.7%), and the AUC decreased to 69.2% (60.4 − 78.0%) (Fig. 3).

Absence of ICP monitoring was associated with mortality (aOR = 7.73, 95% CI: 1.66–35.96, *p* = 0.009) compared with normal ICP, after adjusting for patient demographics, laboratory findings, and CT findings (Supplementary Table S1). There was no independent association between the use of ICP monitoring and PCPC outcomes after adjusting for clinical factors (Supplementary Table S2).

## Discussion

### Performance of clinical prediction rule for raised ICP

In this study, we reported clinical predictors for raised ICP in the pediatric TBI population. However, the clinical prediction models for raised ICP were not sufficiently robust to surrogate for ICP monitoring at both raised ICP thresholds of ICP >20 mmHg or ICP >25 mmHg in our study population. Given the small number of patients (*n* = 151) with ICP monitoring, the generated model for ICP_20mmHg_ achieved a suboptimal sensitivity of 56.0%. This meant that 44% of raised ICP cases would be missed had we used the current model to predict raised ICP in place of ICP monitoring. A previous pediatric TBI study of 199 patients with ICP monitoring using variables within the first 24 h of hospital admission reported a sensitivity of 73% and specificity of 79%.^14^ Of note, we documented clinical variables at presentation, while theirs were within the first 24 h.^14^ The number of patients who underwent ICP monitoring in our cohort was small, limiting the power to derive a high-performing clinical prediction rule.

### Clinical predictors of raised ICP

A longer time to arrival could worsen secondary brain damage, eventually leading to raised ICP.^27,28^ We demonstrated that a longer time to arrival at a hospital was associated with a higher risk of raised ICP_25mmHg_. Compensatory cerebral autoregulation takes place early after TBI.^27,28^ However, delayed presentation to the hospital results in unmitigated secondary brain damage and cerebrovascular dysregulation that could worsen raised ICP.^28,29^ Our findings reinforce the importance of timely intervention of pediatric patients with TBI to prevent secondary brain injury.^29–32^

GCS motor score and skull fracture have previously been reported to be significant factors associated with raised ICP and thus included in our model.^12,13^ GCS motor ≤4 was part of a consensus-based management protocol for raised ICP in the BEST:TRIP study, which compared an ICP monitoring protocol with a non-ICP monitoring consensus-based management protocol for the treatment of severe TBI based on imaging and clinical examination (CREVICE protocol).^12^ They found that using GCS motor ≤4 and other variables guiding treatment for patients without ICP monitoring had similar outcomes as those with ICP monitoring.^12^ Our study found that GCS motor <4 was not independently associated with raised ICP, which could be attributed to differences in the adult and pediatric populations, and a smaller sample size in our study. Signs of basal skull fracture and depressed skull fracture were found to be independently associated with raised ICP in a retrospective cohort adult TBI study attempting to identify clinical predictors of raised ICP.^13^ In our study, we did not find any association between the overall presence of a skull fracture and raised ICP. Future larger prospective studies should investigate the association specifically between basal skull fracture and raised ICP in pediatric TBI.^33,34^

### Association between ICP monitoring and clinical outcomes

Our MLR models found that patients not monitored for raised ICP were 7.73 times more likely to have in-hospital mortality compared with those with normal ICP and 3 times more likely than those with raised ICP. Our findings are congruent with previous studies that reported reduced mortality with the usage of ICP monitoring in pediatric patients with TBI.^35–37^ Contrary to our findings, a retrospective observational study of 3808 pediatric patients with TBI reported that ICP monitoring may be associated with mortality.^37^ The children in their study who underwent ICP monitoring were older, had a higher injury severity score, and a larger proportion of patients who suffered TBI from road traffic accidents, compared with the group without ICP monitoring.^38^ In contrast, our three groups of raised ICP, no raised ICP, and no ICP monitoring had similar ages and mechanisms of injury. Our findings support the current recommendation by the BTF in using ICP monitoring for not just severe pediatric TBI but potentially also among children with moderate TBI. Future pediatric TBI studies should incorporate longer-term functional assessments and stratification of TBI severity when assessing functional outcomes in relation to raised ICP.

### Strengths and limitations

Our study comprised a multinational cohort across Asia and Latin America, with relevance to the global population. Our primary outcomes explored in-hospital mortality and functional outcomes, and our findings have the potential to guide clinicians in prognostication and communication with the families of patients.

As an observational study, TBI management practices were not standardized across participating sites, which may have led to confounding in our analysis. Also, we were not able to obtain details of pre-hospital management, as well as the extent of neurorehabilitation post-injury. Among children with external ventricular drains, we were unable to provide further details on cerebrospinal fluid drainage. We also recognize limitations in neurological and radiological monitoring. We did not perform serial GCS measurements beyond the presenting and worst scores in the first 24 h. We only obtained the initial CT at presentation, which precluded subsequent radiological assessments. We were unable to obtain standardized CT scoring systems (Marshall or Rotterdam scores) in our study. Future prospective multicenter studies could investigate longer-term outcomes. The actual number of patients with ICP monitoring was small, limiting our ability to identify clinical predictors and build a clinical prediction model for raised ICP. Future studies that investigate clinical predictors of raised ICP should increase collaboration with other pediatric hospitals to generate sufficient statistical power. Nevertheless, we demonstrated that many children with moderate-to-severe TBI in our regions do not routinely receive ICP monitoring.

## Conclusion

Our study demonstrates the utility of ICP status in clinical practice. We reported on clinical predictors for raised ICP. Unfortunately, the model predicting raised ICP in our study was not robust enough to surrogate for ICP monitoring. Future large-scale prospective studies should stratify clinical risk prediction for raised ICP by specific intracranial pathologies.

## Transparency, Rigor, and Reproducibility

The study was not formally registered and is a secondary analysis of an ongoing TBI collaborative research study. The analysis plan was not formally preregistered but the principal investigator certifies that the analysis was prespecified. A total sample size of 150 subjects was planned to allow a model development set of 75 participants each. Eight hundred and three potential participants were screened, 95 patients were excluded due to exclusion criteria and inadequate data, and adequate data were obtained from 706 patients. Data analysis was performed by investigators who were aware of the relevant characteristics of the participants. PACCMAN sites collected data retrospectively from 2014 to 2017 and then prospectively from 2018 to 2022. LARed sites collected prospective data from 2021 to 2022. Data were collected in a standardized electronic form (REDCap, Vanderbilt University) with fields defined *a priori*. The data were analyzed using SPSS v28 (IBM Corp., Released 2021. IBM SPSS Statistics for Macintosh, Version 28.0. Armonk, NY: IBM Corp., Chicago) and STATA v18 (StataCorp., 2023. Stata Statistical Software: Release 18. College Station, TX: StataCorp LLC). The key inclusion criteria and outcome evaluations are based on established standards. Statistical analysis and review were performed by B.Z.H.J. with qualifications including an MD and MSc in Clinical Epidemiology and a PhD in Health Economics. CIs have been reported in the abstract for primary outcomes and main text for all outcomes. De-identified data from this study are not available in a public archive. De-identified data from this study will be made available (as allowable according to Institutional Review Board standards) by emailing the corresponding author. There is no analytic code associated with this study. The authors agree to publish the full content of the article on Mary Ann Liebert, Inc. under an appropriate license.

## Abbreviations Used

AUC: Area under the curve
BTF: Brain Trauma Foundation
CT: Computed tomography
GCS: Glasgow Coma Scale
ICP: Intracranial pressure
IQR: Interquartile range
LARed: Red Colaborativa Pediátrica de Latinoamerica
MLR: Multivariable logistic regression
PACCMAN: Pediatric Acute & Critical Care Medicine Asian Network
PCPC: Pediatric Cerebral Performance Category
ROC: Receiver operating characteristic curve
SDI: Sociodemographic Index
TBI: Traumatic brain injury
