# Supplementary material for: Identifying Clinical Predictors of Raised Intracranial Pressure in Pediatric Traumatic Brain Injury—A Multinational Initiative
Source: Neurotrauma Rep. 2025 Sep 9;6(1):778–89. doi: 10.1177/2689288X251370703 (PMC12528845; doi:10.1177/2689288X251370703)
Supplement: Supplementary Table S1 [file 2689288x251370703_supplementary_table_s1.docx]

**Supplementary Table 1: Multivariable logistic regression predicting for mortality, n=706**

|  | Unadjusted odds ratio with 95% CI | p-value | Adjusted Odds ratio with 95% CI | p-value |
| --- | --- | --- | --- | --- |
| ICP status |  |  |  |  |
| Normal ICP (reference) | 1.00 | - | 1.00 | - |
| Raised ICP | 2.12 (0.51-8.80) | 0.302 | 2.34 (0.40- 13.55) | 0.344 |
| No ICP monitoring | 3.69 (1.13-12.00) | 0.030* | 7.73 (1.66- 35.96) | 0.009* |
| Hyperosmolar therapy^1^ | 2.54 (1.42-4.54) | 0.002* | 2.68 (1.18- 6.10) | 0.018* |
| Intubation within 24 hours | 12.80 (3.11-52.68) | <0.001* | 7.39 (1.51- 36.06) | 0.013* |
| Male sex | 0.69 (0.43-1.10) | 0.113 | 0.28 (0.20- 0.72) | 0.003* |
| Low-Middle SDI | 0.72 (0.44-1.16) | 0.175 | 0.39 (0.21- 0.75) | 0.004* |
| Travelling by own transport | 0.40 (0.19-0.85) | 0.018 | 0.52 (0.20- 1.37) | 0.186 |
| Injury type |  |  |  |  |
| Traffic accident (Reference) | 1.00 | - | 1.00 | - |
| Fall | 0.64 (0.39-1.05) | 0.074 | 0.85 (0.43- 1.66) | 0.629 |
| Child abuse | 0.76 (0.26-2.23) | 0.612 | 0.44 (0.10- 1.89) | 0.273 |
| Other | 0.43 (0.13-1.45) | 0.174 | 0.39 (0.06- 2.45) | 0.318 |
| GCS motor <4 | 7.30 (4.22-12.65) | <0.001* | 6.41 (3.21- 12.80) | <0.001* |
| Multiple trauma | 2.86 (1.73-4.71) | <0.001* | 1.50 (0.73- 3.08) | 0.266 |
| Thrombocytopenia^2^ | 2.85 (1.61-5.02) | <0.001* | 1.38 (0.63- 3.00) | 0.417 |
| Leucocytosis^3^ | 0.47 (0.28-0.79) | 0.004* | 0.42 (0.21- 0.85) | 0.016* |
| Dysnatraemia^4^ | 3.47 (2.12-5.70) | <0.001* | 3.10 (1.57- 6.13) | 0.001* |
| Coagulopathy^5^ | 4.19 (2.40-7.32) | <0.001* | 3.07 (1.51- 6.13) | 0.002* |
| Skull fracture on CT | 0.77 (0.49-1.23) | 0.272 | 0.69 (0.36- 1.31) | 0.257 |
| Midline shift on CT | 2.60 (1.56-4.32) | <0.001* | 3.82 (1.96- 7.44) | <0.001* |

CI=Confidence Interval

ICP=Intracranial Pressure

SDI=Social Demographic Index

GCS=Glasgow Coma Scale

CT= Computed Tomography

^1^Hyperosmolar therapy was defined as the use of 3% hypertonic saline or 20% mannitol within the first 24 hours of hospital admission.

^2^Thrombocytopenia was defined as a platelet count <150x10^9^/L.

^3^Leucocytosis was defined as a leucocyte count >11.0x10^9^/L.

^4^Dysnatraemia was defined as sodium values <135 or >145mmol/L.

^5^Coagulopathy was defined as prothrombin time >15s, partial thromboplastin time >40s, or an international normalised ratio >1.2.
