# Supplementary material for: Identifying Clinical Predictors of Raised Intracranial Pressure in Pediatric Traumatic Brain Injury—A Multinational Initiative
Source: Neurotrauma Rep. 2025 Sep 9;6(1):778–89. doi: 10.1177/2689288X251370703 (PMC12528845; doi:10.1177/2689288X251370703)
Supplement: Supplementary Table S2 [file 2689288x251370703_supplementary_table_s2.docx]

**Supplementary Table 2: Multivariable logistic regression predicting for poor PCPC outcomes, n=615**

|  | Unadjusted odds ratio with 95% CI | p-value | Adjusted Odds ratio with 95% CI | p-value |
| --- | --- | --- | --- | --- |
| ICP status |  |  |  |  |
| Normal ICP (reference) | 1.00 | - | 1.00 | - |
| Raised ICP | 1.58 (0.80-3.16) | 0.196 | 1.90 (0.89-4.06) | 0.098 |
| No ICP monitoring | 0.50 (0.29-0.87) | 0.014* | 0.80 (0.43-1.46) | 0.462 |
| Intubation within 24 hours | 5.35 (2.81-10.20) | <0.001* | 3.38 (1.63-6.94) | 0.001* |
| Low-Middle SDI | 1.73 (1.19-2.51) | 0.344 | 1.49 (0.99-2.27) | 0.059 |
| Injury type |  |  |  |  |
| Traffic accident (Reference) | 1.00 | - | 1.00 | - |
| Fall | 0.63 (0.42-0.94) | 0.022 | 0.67 (0.43-1.06) | 0.087 |
| Child abuse | 1.36 (0.63-2.94) | 0.441 | 1.09 (0.44-2.68) | 0.849 |
| Other | 0.73 (0.33-1.60) | 0.428 | 0.86 (0.36-2.03) | 0.726 |
| GCS motor <4 | 3.62 (2.46-5.31) | 0.114 | 2.93 (1.92-4.47) | <0.001* |
| Thrombocytopenia^1^ | 1.89 (1.10-3.28) | 0.022 | 1.83 (0.96-3.49) | 0.066 |
| Leucocytosis^2^ | 1.51 (0.88-2.57) | 0.135 | 1.73 (0.95-3.15) | 0.071 |
| Dysnatraemia^3^ | 1.43 (0.88-2.31) | 0.146 | 1.30 (0.75-2.24) | 0.350 |
| Coagulopathy^4^ | 1.50 (1.04-2.18) | 0.031 | 1.37 (0.90-2.09) | 0.146 |
| Skull fracture on CT | 0.81 (0.56-1.18) | 0.276 | 0.69 (0.45-1.05) | 0.087 |
| Midline shift on CT | 1.32 (0.85-2.04) | 0.211 | 1.28 (0.78-2.12) | 0.329 |

CI=Confidence Interval

ICP=Intracranial Pressure

SDI=Social Demographic Index

GCS=Glasgow Coma Scale

CT= Computed Tomography

^1^Thrombocytopenia was defined as a platelet count <150x10^9^/L.

^2^Leucocytosis was defined as a leucocyte count >11.0x10^9^/L.

^3^Dysnatraemia was defined as sodium values <135 or >145mmol/L.

^4^Coagulopathy was defined as prothrombin time >15s, partial thromboplastin time >40s, or an international normalised ratio >1.2.
